# Antioxidant Pre-Treatment Reduces the Toxic Effects of Oxalate on Renal Epithelial Cells in a Cell Culture Model of Urolithiasis

**DOI:** 10.3390/ijerph14010109

**Published:** 2017-01-23

**Authors:** Tomislav Kizivat, Martina Smolić, Ivana Marić, Maja Tolušić Levak, Robert Smolić, Ines Bilić Čurčić, Lucija Kuna, Ivan Mihaljević, Aleksandar Včev, Sandra Tucak-Zorić

**Affiliations:** 1 Clinical Hospital Osijek, Josipa Huttlera 4, HR-31000 Osijek, Croatia; tomislavkizivat@gmail.com (T.K.); Ivana.Maric@mefos.hr (I.M.); robert.smolic@mefos.hr (R.S.); ibcurcic@mefos.hr (I.B.Č.); imihaljevic@mefos.hr (I.M.); avcev@mefos.hr (A.V.); 2 Faculty of Medicine Osijek, Josip Juraj Strossmayer University of Osijek, Cara Hadrijana 10, HR-3100 Osijek, Croatia; mtolusic@mefos.hr (M.T.L.); kunalucija@gmail.com (L.K.); atucak@mefos.hr (S.T.-Z.)

**Keywords:** urolithiasis, vitamin E, l-arginine, Madin-Darby canine kidney cells, LLC-PK1 cells

## Abstract

Urolithiasis is characterized by the formation and retention of solid crystals within the urinary tract. Kidney stones are mostly composed of calcium oxalate, which predominantly generates free radicals that are toxic to renal tubular cells. The aim of the study is to explore possible effects of antioxidant pre-treatment on inhibition of oxidative stress. Three cell lines were used as in vitro model of urolithiasis: MDCK I, MDCK II and LLC-PK1. Oxidative stress was induced by exposure of cells to sodium oxalate in concentration of 8 mM. In order to prevent oxidative stress, cells were pre-treated with three different concentrations of l-arginine and vitamin E. Oxidative stress was evaluated by determining the expression of superoxide dismutase (SOD), osteopontin (OPN), and by the concentration of glutathione (GSH). In all three cell lines, pre-treatment of antioxidants increased cell survival. Positive correlation of SOD and OPN expression as well as GSH concentration was observed in all groups of cells. Our results indicate that an antioxidant pre-treatment with l-arginine and vitamin E is able to hamper oxalate-induced oxidative stress in kidney epithelial cells and as such could play a role in prevention of urolithiasis.

## 1. Introduction

Urolithiasis is characterized by the creation of solid deposits inside of the urinary tract. It continues to be an important factor in chronic renal disease leading to chronic tubular interstitial nephritis, which is involved in 15%–20% of end-stage chronic kidney insufficiency [1]. It is expected that 11% of men and 5.6% of women in United States will develop solid deposits in their urinary tract by the age of seventy [2]. Moreover, in the last forty years, the prevalence and incidence of urolithiasis has been increasing [3], mostly due to dietary [4] and climate changes [5]. Recurrence of urolithiasis is high, with rates of 40% within 5 years, and 75% within 10 years [3].

With the global warming debate, much attention has been given to the changes in human health caused by increased temperatures [6], as well as about the increase of incidence of nephrolithiasis due to climate changes [5]. The mechanism of higher temperatures causing urolithiasis is related to heat-induced sweating, leading to reduction in urinary volume thus concentrating relatively insoluble salts [7]. Dietary changes have led to an increase in body weight, which had also been related to the increased risk of stone formation [4]; moreover, bariatric surgery has been connected to increased risk of oxalate stone formation [8].

Although great advances have been made in the treatment of urolithiasis by extracorporeal shock wave lithotripsy, urolithiasis remains a significant health burden. One of the reasons accounting for this is that the molecular mechanism of stone formation is still not fully elucidated.

Four possible models of stone formation in humans and animals have been identified: growth over Randall’s plaque, growth over Bellini duct plugs, formation of microliths within inner medullary collecting ducts (which has thus far only been seen in cystinuria) and formation in free solution within the calyces or renal collecting systems [9]. For the first two models, an important step in the formation of the stone is endothelial damage [9,10,11]. Researchers in pathogenesis of calcium oxalate stone formation have shown that oxalate-induced generation of oxidative stress could play a significant role in endothelial damage [12,13]. Increased quantities of oxalate could trigger the production of reactive oxygen species in kidneys leading to endothelial injury and apoptosis, which could provide sites for crystal formation [14,15]. Moreover, cell degradation results in the generation of numerous membrane vesicles, which are effective crystal nucleators, promoting nucleation at low supersaturation, and furthermore promote crystal-cell interactions [12,16].

It has also been shown that interaction of epithelial cells with oxalate stimulates osteopontin (OPN) expression [17], which has been shown recently to play an important role in kidney stone formation [18,19], although its role is still not completely clear. However, not only oxidative stress, but also nitrosative stress can have a role in creation of urinary stones by nitration of Tamm-Horsfall glycoprotein (THP), which can increase the risk of crystallization of urine [20].

It has been suspected that antioxidants like vitamin E and l-arginine, which can also prevent nitrosative stress [21], can have a role in preventing cell damage caused by free radicals. It was shown that antioxidants enhance expression of antioxidant enzyme superoxide dismutase (SOD) in a calcium oxalate crystal [22] and ethylene glycol-induced [23] model of urolithiasis.

Therefore, the aim of our study was to evaluate the toxic effect of oxalate on renal epithelial cells and to explore possible effects of antioxidants on oxalate in a cell culture model of urolithiasis.

## 2. Materials and Methods

Chemicals. Sodium oxalate (purified to ≥99%), l-arginine and vitamin E were purchased from Sigma Chemicals (St. Louis, MO, USA).

Cell Lines and Cell Culture. Madin-Darby canine kidney cells subtype I (MDCK I), subtype II (MDCK II) and LLC-PK1 cell lines were used. Parent MDCK cell type was isolated from healthy full grown cocker spaniels, and subsequently several subtypes were isolated from the parent cell line [24]. MDCK I cells can be described as collector, and MDCK II as proximal tubules of the kidney [25]. An LLC-PK1 cell type was isolated from a kidney of a healthy, male, Hampshire pig, and shows the characteristics of proximal kidney tubules [26]. All cell lines were a generous gift from Professor Carl Verkoelen’s laboratory at the Urology Clinic of Erasmus Medical Center in Rotterdam, The Netherlands. Cells were sub-cultivated in 10 cm dishes in Dulbecco’s modified eagle medium (DMEM) supplemented with 10% fetal bovine serum (FBS) and 1% antibiotic/antimycotic solution (Thermo Fisher Scientific Inc., Waltham, MA, USA). Cells were grown at 37 °C in a humidified atmosphere of 5% CO_2_ (*v*/*v*) in air.

To establish sodium oxalate toxicity, cells were maintained in DMEM without FBS.

Antioxidant Protection Assays. To determine protective effects of antioxidants, l-arginine (at concentration 0.5, 0.1 and 0.05 ng/mL) and vitamin E (at concentrations 25, 15 and 5 µM) were used. l-arginine was dissolved at appropriate concentrations in phosphate buffer saline (PBS), and vitamin E was dissolved according to previously published protocol [27]. To induce oxidative stress 8 mM sodium oxalate was added to the medium without FBS as our previous work showed that this concentration of sodium oxalate causes death in 50% of cells if treated overnight (data not shown).

Cells were plated at density 4 × 10^5^ cells/mL in 6-well plates and exposed to the antioxidant in appropriate concentrations. Cells were grown overnight and on the second day the medium was changed to DMEM without FBS and 8 mM sodium oxalate was added. Cells were grown overnight, and on the third day trypsinized to determine cell survival by trypan blue exclusion and Neubauer Hemocytometer counting. Three controls were used; untreated cells, cells treated only with antioxidant (0.1 ng/mL of l-arginine or 15 µM of vitamin E) and cells treated with sodium oxalate only. Results were expressed as percentage compared to untreated controls. Experiments were done in triplicates.

Total RNA Isolation and Reverse Transcription Polymerase Chain Reaction (RT-PCR) Analysis. To evaluate the expression of extracellular superoxide dismutase (SOD) and osteopontine (OPN), total RNA from the cells was isolated on day three of the experiment using RNeasy Mini Kit (Qiagen, Hilden, Germany). First strand cDNA was synthesized by manufacturer’s protocol (PrimeScript First StrandcDNASynthesis Kit, Takara Bio, Otshu-Shi, Japan). The synthesized cDNA was amplified using specific primer sequences as follows: SOD (sense 5′-ATGGTGGCCTTCTTGTTCTGC-3′, antisense 5′-GTGCTGTGGGTGCGGCACACC-3′); OPN (sense 5′-CCATGAGACTGGCAGGGTT-3′, antisense 5′-GGAACTGTGGTTTTGCCTCT-3′) and LDHA (sense 5′-TAATGAAGGACTTGGCAGATGAACT-3′, antisense 5′-ACGGCTTTCTCCCTCTTGCT-3′) [28]. PCR conditions were: for SOD and OPN denaturation at 94 °C for 3 min, annealing at 59 °C for 45 s, elongation at 72 °C for 1 min in 30 cycles; for LDHA denaturation at 94 °C for 3 min, annealing at 57 °C for 45 s, elongation at 72 °C for 1 min in 30 cycles. The PCR products were run on 0.8% agarose gel, stained with SYBR Safe DNA Gel Stain (Thermo Fischer Scientific, Waltham, MA, USA), visualized and semi quantified by Image Lab software (version 5.2.1 build 11, BioRad, Hercules, CA, USA). Results are shown as percentages compared to untreated cells.

Glutathione (GSH) Assay. To determine concentrations of GSH, cells were prepared per above described protocol. On the third day, cells were detached by rubber policeman and cell numbers were normalized to concentration of 1 × 10^8^ cells per milliliter. Concentration of GSH was measured spectrophotometrically by commercially available kit (Glutathione Assay Kit, Sigma-Aldrich, St. Louis, MO, USA) per manufacturer’s protocol. Results are shown in nanomoles per milliliter of sample.

Statistical Analyses. All numerical data were expressed as means ± SD. Statistical analyses were performed using Student’s *t*-test; *p*-values of <0.05 were considered statistically significant.

## 3. Results

**Figure 1:**
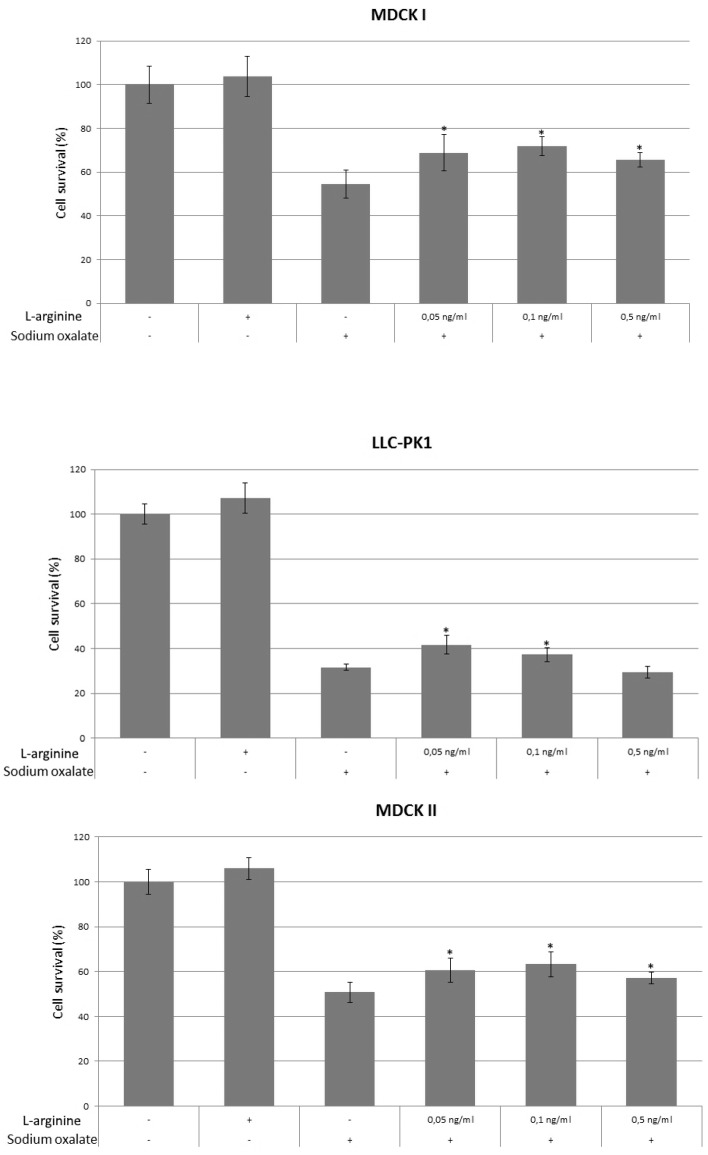
Effects of l-arginine on cell survival in MDCK I, MDCK II and LLC-PK1 cell lines. Three controls of untreated cells, cells treated with l-arginine only and cells treated with sodium oxalate only were used. Increasing concentrations of l-arginine (0.05, 0.01 and 0.5 ng/mL) were used for pretreatment of cells prior to exposure to sodium oxalate to evaluate the effects on cell survival. The values are presented as means ± standard deviation. Plus (+) and minus (−) sign underneath the x-axis indicate addition of l-arginine and sodium oxalate, respectively. Bars assigned with asterisk (*) are statistically significantly different (*p* < 0.05) compared to control treated with sodium oxalate only, as determined with Student’s *t*-test. The data shown are representative of at least three independent experiments.

To show effects of l-arginine on cell survival, cells were pretreated with l-arginine in appropriate concentrations overnight, on the second day the medium was changed and sodium oxalate was added and treated overnight. On the third day, cell survival was determined, and compared to controls. The results are shown in Figure 1. In all cell lines, 0.05 and 0.1 ng/mL of l-arginine showed statistically significant higher survival compared to oxalate-alone treated cells. In MDCK I cell line survival of oxalate-alone treated cells was 55% compared to 0.05 ng/mL l-arginine pretreated cells 69% (*p* = 0.03) and 0.1 ng/mL l-arginine pretreated cells 72% (*p* = 0.01). In MDCK II cell line, survival of oxalate-alone treated cells was 51% compared to 0.05 ng/mL l-arginine pretreated cells 61% (*p* = 0.04) and 0.1 ng/mL l-arginine pretreated cells 63% (*p* = 0.02). In LLC-PK1 cell line, survival of oxalate-alone treated cells was 32% compared to 0.05 ng/mL l-arginine pretreated cells 42% (*p* = 0.003) and 0.1 ng/mL l-arginine pretreated cells 37% (*p* = 0.01). l-arginine, 0.5 ng/mL, showed statistically higher survival in MDCK I (66% compared to oxalate-alone treated cells 55%, *p* = 0.03) and MDCK II cell lines (57% compared to oxalate-alone treated cells 51%, *p* = 0.04), but not in the LLC-PK1 line. In MDCK I and MDCK II cell lines the peak survival was shown at 0.1 ng/mL, peak survival in LLC-PK1 was at 0.05 ng/mL indicating that the effects of l-arginine were similar in distal and proximal tubules, but had different effects depending on species origin of the cells. There was no statistically significant difference between untreated cells and cells treated only with l-arginine in all cell lines used.

**Figure 2:**
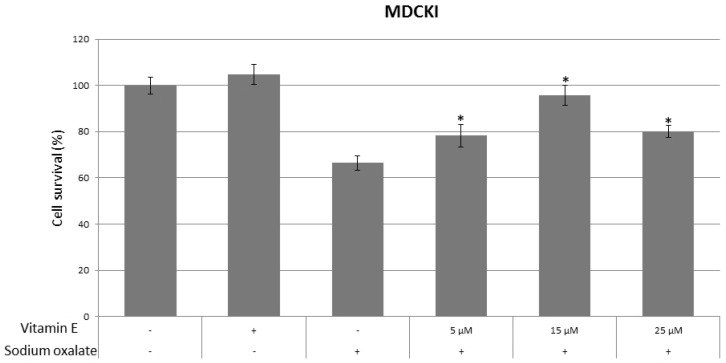

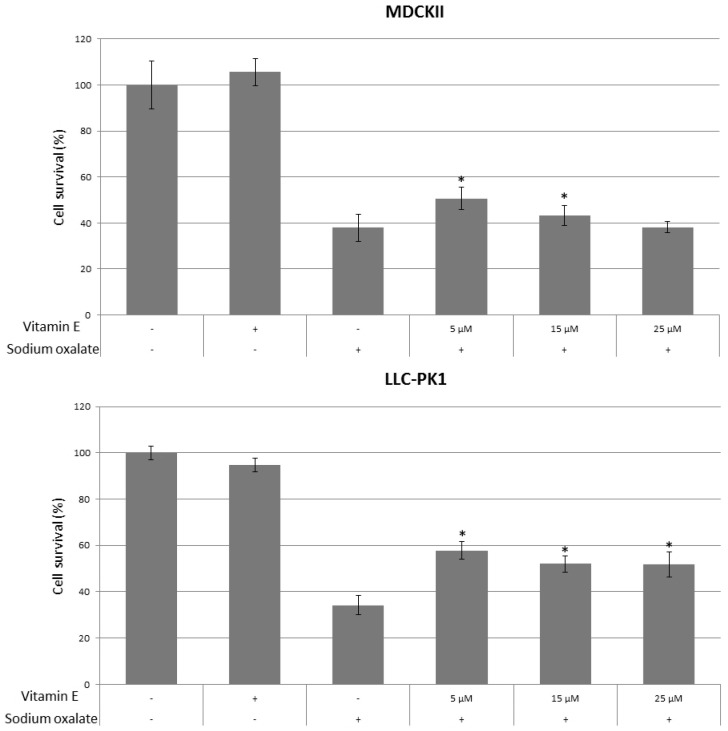
Effects of vitamin E on cell survival in MDCK I, MDCK II and LLC-PK1 cell lines. Three controls of untreated cells, cells treated with vitamin E only and cells treated with sodium oxalate only were used. Increasing concentrations of vitamin E (5, 15 and 25 µM) were used for pretreatment of cells prior to exposure to sodium oxalate to evaluate the effects on cell survival. The values are presented as means ± standard deviation. Plus (+) and minus (−) sign underneath the x-axis indicate addition of vitamin E and sodium oxalate, respectively. Bars assigned with asterisk (*) are statistically significantly different (*p* < 0.05) compared to control treated with sodium oxalate only, as determined by Student’s *t*-test. The data shown are representative of at least three independent experiments.

Effects of vitamin E on cell survival are shown in Figure 2. Cells were pretreated with appropriate concentrations of vitamin E and plated in six well dishes overnight, on the second day medium was changed and sodium oxalate was added and treated overnight, on the third day cell survival was determined, and compared to cells treated only with sodium oxalate. Cells treated with vitamin E and subsequently treated with sodium oxalate had a higher survival than cells treated only with sodium oxalate. All cell lines treated with 5 and 15 µM of vitamin E had a statistically significant higher survival rates compared to oxalate-alone treated controls. In MDCK I cell line, oxalate-alone treated cells had 66% survival compared to 5 µM vitamin E pretreated cells which had 78% survival (*p* = 0.009) and 15 µM vitamin E pretreated cells which had 95% survival (*p* = 0.0001). In MDCK II cell line, oxalate-alone treated cells had 38% survival compared to 5 µM vitamin E pretreated cells which had 50% survival (*p* = 0.0004) and 15 µM vitamin E pretreated cells which had 43% survival (*p* = 0.03). In LLC-PK1 cell line, oxalate-alone treated cells had 34% survival compared to 5 µM vitamin E pretreated cells which had 58% survival (*p* = 0.0007) and 15 µM vitamin E pretreated cells which had 52% survival (*p* = 0.002). But only in MDCK I (80% compared to oxalate-alone treated cells 66%, *p* = 0.0008) and LLC-PK1 (52% compared to oxalate-alone treated cells 34%, *p* = 0.002) cell lines, cells treated with 25 µM had statistically significant higher rates of survival. The peak of cell survival in MDCK I cell line was at 15 µM, while for MDCK II and LLC-PK1 cell lines, it occurred at a lower concentration of 5 µM. This indicates that the effects of vitamin E is more related to the location of tubules than its species origin. There was no statistically significant difference between untreated cells and cells treated only with vitamin E in all cell lines used.

**Figure 3:**
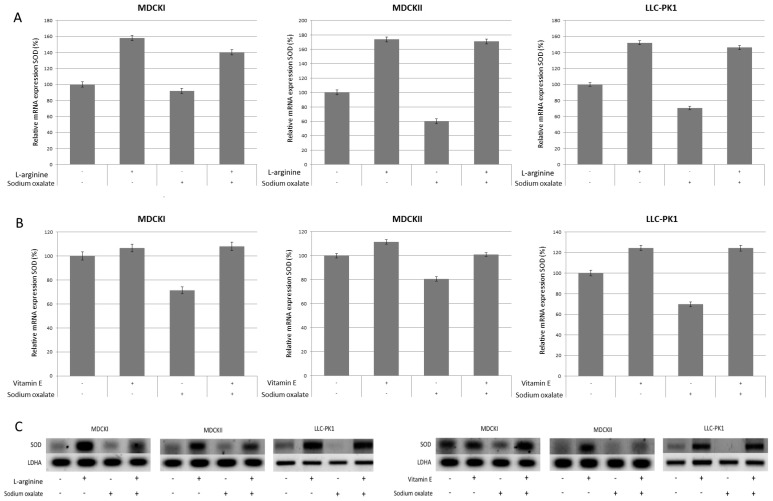
Effects of l-arginine and Vitamin E pretreatment on the expression on superoxide dismutase (SOD) gene in MDCK I, MDCK II and LLC-PK1 cell lines. (**A**,**B**) panels: SOD expression was inhibited in controls treated with sodium oxalate only, and was promoted in controls treated with antioxidants (l-arginine and vitamin E) only. SOD was stronger expressed in cells pretreated with antioxidant and then exposed to sodium oxalate compared to control cells treated with sodium oxalate only, but somewhat weaker expressed compared to cells treated with antioxidant only. The gene expression analysis was done by RT-PCR and obtained results were semi-quantified by ImageLab software. The values are represented as means ± SD. The data shown are representative of at least three independent experiments. Panel (**C**): Representative figures of Southern blot analysis of SOD expression compared to LDHA expression.

Antioxidants were previously shown to promote expression of SOD in renal epithelial cell lines. Therefore, we analyzed mRNA expression of SOD in all three cell lines for l-arginine and vitamin E, and sodium oxalate, as shown in Figure 3. Results are shown for l-arginine at concentration of 0.1 ng/mL and vitamin E at concentration of 15 µM. As expected, SOD expression, compared to untreated controls, was lower in cells treated only with sodium oxalate (71%–90% for MDCK I, 60%–71% for MDCK II and 70% for LLC-PK1), and higher in cells treated only with antioxidants. In MDCK I cells, SOD expression for l-arginine only treated cells compared to untreated cells was 158% while for vitamin E only treated cells was 106%. MDCK II cells had SOD expression for l-arginine-only treated cells of 174% compared to untreated controls and 111% for vitamin E. SOD expression in LLC-PK1 cells treated only with l-arginine compared to untreated controls was 152% and for vitamin E 124%. In cells pretreated with antioxidants and exposed to sodium oxalate, SOD expression was higher than in control cells treated only with sodium oxalate and untreated cells but it was lower than cells treated only with antioxidant. As shown in Figure 3, in MDCK I cells SOD expression for l-arginine pre-treated cells was 140% and for vitamin E 108%; similarly, in MDCK II cells for l-arginine 143% and for vitamin E 100%, while SOD expression in LLC-PK1 cells for l-arginine pre-treated cells was 146% and vitamin E 124%.

**Figure 4:**
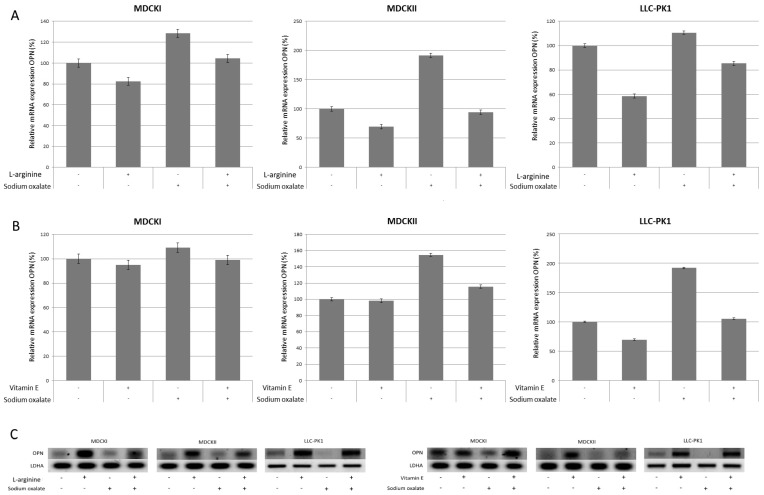
Effects of l-arginine and vitamin E pretreatment on the expression on osteopontin (OPN) gene in MDCK I, MDCK II and LLC-PK1 cell lines. (**A**,**B**) panels: OPN expression was promoted in controls treated with sodium oxalate only, and was inhibited in controls treated with antioxidants (l-arginine and vitamin E) only. OPN gene expression was suppressed in cells pretreated with antioxidant and later exposed to sodium oxalate when compared to OPN gene expression in control cells treated with sodium oxalate only, but somewhat higher expressed than in cells treated with antioxidant only. The gene expression analysis was done by reverse transcriptase PCR and semi quantified by ImageLab software. The values are represented as means ± SD. The data shown are representative of at least three independent experiments. (**C**) panel: Representative figures of Southern blot analysis of OPN gene expression compared to LDHA gene expression.

OPN expression was increased in cells treated with sodium oxalate only, and was decreased in cells treated with antioxidant only. In cells pretreated with antioxidant and subsequently exposed to sodium oxalate, OPN expression was decreased compared to cells treated only with sodium oxalate, but was increased compared to cells treated with antioxidant only, as shown in Figure 4. Results are shown for l-arginine at concentration of 0.1 ng/mL and vitamin E at concentration of 15 µM. OPN expression in MDCK I cells was: for oxalate-only treated cells 128%, for l-arginine only treated cells 82% and for vitamin E only treated cells 95%; for l-arginine pre-treated and oxalate treated cells 104% and for vitamin E pretreated and subsequently oxalate treated cells 99%. Similarly, OPN expression in MDCK II cells was: for oxalate only treated cells 154%, for l-arginine only treated cells 70% and for vitamin E only treated cells 98%; for l-arginine pre-treated and subsequently oxalate treated cells 94% and for vitamin E pretreated and subsequently oxalate treated cells 119%. In LLC-PK1 cells OPN expression was similar: for oxalate only treated cells 110%, for l-arginine only treated cells 58% and for vitamin E only treated cells 69%; for l-arginine pre-treated and oxalate treated cells 85% and for vitamin E pretreated and subsequently oxalate treated cells 105%.

**Figure 5:**
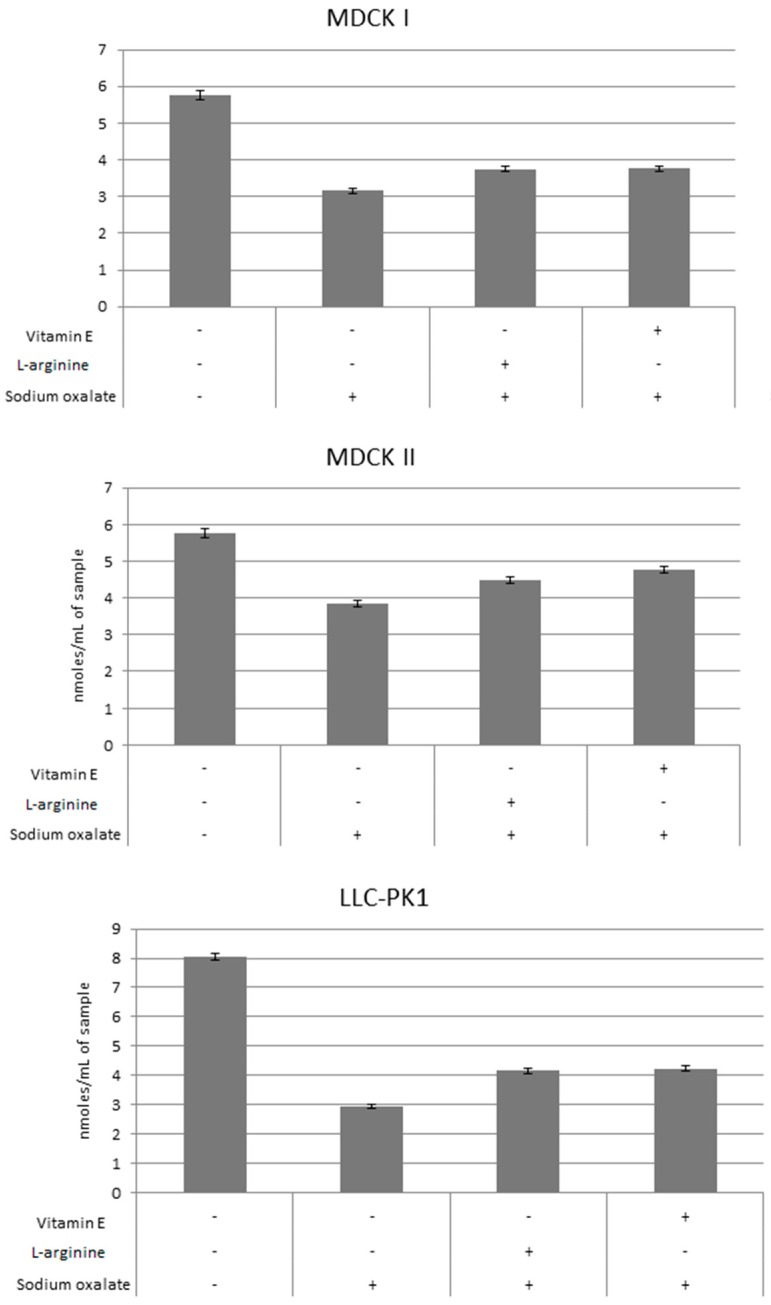
Effects of l-arginine and vitamin E pretreatment on the levels of GSH in MDCK I, MDCK II and LLC-PK1 cell lines. GSH levels were lower in oxalate-only treated cells compared to untreated controls, cells pretreated with l-arginine and vitamin E showed recovery of GSH levels. GSH measurement was done using spectrophotometry. The values are represented as means ± SD. The data shown are representative of at least three independent experiments.

To evaluate cellular redox tone, GSH levels were measured in all three cell lines after treatment with oxalate only and after pretreatment with antioxidants and subsequent treatment with oxalate, as shown in Figure 5. Results are shown for l-arginine at concentration 0.1 ng/mL and vitamin E at concentration 15 µM. Untreated cells were used as a control and GSH levels measured. In all three cell lines, treatment with oxalate showed significant decrease of GSH levels compared to untreated controls. Pretreatment with l-arginine and vitamin E caused significant recovery of GSH levels compared to cells treated with oxalate only. There was no significant difference in GSH levels between cells pretreated with l-arginine and vitamin E, although a tendency of higher GSH levels was shown in cells pretreated with vitamin E.

## 4. Discussion

Nephrolithiasis has multifactorial causes, including diet, climate and genetic causes. One of the important factors is supersaturation of urine by salts, including oxalate, causing renal epithelial cell injury [29]. Recently, dietary agents have been proposed as suitable treatment or prevention of urolithiasis, in part because of their antioxidant effects [22,30]. Despite their other benefits, the potential of l-arginine and vitamin E as agents for prevention of urolithiasis has not yet been explored.

Increased saturation of urine by oxalate causes damage to epithelial cells, thus causing cell damage and death, increasing oxalate crystal binding to cells and forming nucleus for further crystallization [31,32,33]. By pretreating renal epithelial cells with antioxidants we showed that cell survival was increased, thus indicating that they could be used as a prevention agent for urolithiasis. By using three different cell lines originating from two different species (pig and dog) our results indicate that vitamin E has the same effect on epithelial cell lines of same location in the kidney in different species. In contrast, l-arginine had the same effect in proximal and collection tubules of the same species (dog), but had different effect on proximal tubules of pig kidney cell line. In LLC-PK1 cell line, cell survival was smaller for the same concentrations of l-arginine in comparison to MDCK I and II cell lines with peak of the effects shifted toward smaller concentrations of the antioxidant. These results are comparable to previous work done on kidney epithelial cell lines, which had shown dose dependence of antioxidant effect in reducing oxidative stress [34,35]. This shows that further studies are needed to determine what effect it would have on human kidney epithelial cells.

Superoxide dismutases (SOD) are very important enzymatic antioxidants that rapidly catalyze the dismutation of superoxide, and promote its removal [36]. In the case of a deficiency in SOD the superoxide preferentially reacts with NO and produces peroxynitrite, a powerful oxidizing and nitrating agent that can directly damage proteins, lipids and DNA [37,38]. In previous studies, it had been shown that green tea extract significantly increases SOD activity and expression in ethylene glycol hyperoxaluric rat model [23]. In the current study, we have shown that vitamin E and l-arginine increased expression of SOD in all three cell lines exposed to oxalate. These effects of vitamin E and l-arginine could block pathways that could lead to crystal formation in the kidney.

Osteopontin is an important component of stone matrix [39]. It is believed to be involved in many pathogenic and physiologic processes, among others in kidney stone formation and renal injury [18,19,22,40]. In normal animal and human kidneys, it is mostly expressed in specific sites like the loop of Henle and distal nephrons; however, it can be significantly upregulated in almost all tubular segments after renal injury, which is especially seen with kidney stones [39,41,42]. It is associated with renal stone formation; however, its role is still controversial. It is not clear if OPN acts as a promoter or inhibitor of stone formation [39]. OPN may act as modulator of calcium oxalate crystallization, associated in nucleation and growth of the crystals [42,43]. In our study, significant downregulation of OPN expression in cells pretreated with antioxidants compared to cells treated with oxalate only was shown, indicating that used antioxidants decrease cellular damage and could possibly have a role in prevention of urolithiasis.

Under physiological conditions, cells are equipped with numerous antioxidant systems, among others enzymatic (above discussed SOD) and non-enzymatic (GSH, vitamins E, A and C) to limit the effects of free radicals [44]. Up to a certain point, they can control the damage caused by free radicals, when this point is exceeded, cells are faced with oxidative stress, and addition of external antioxidants is necessary to maintain cell viability [45,46]. Our data determining GSH levels and SOD expression clearly show that pretreatment with vitamin E and L-arginine of cells later exposed to oxalate reduces oxidative stress.

## 5. Conclusions

In conclusion, vitamin E and l-arginine showed a significant increase in renal epithelial cell survival in all three cell lines, although there was some difference in concentrations required for the same effect in different cell lines. They also increased expression of SOD, concentration of GSH and decreased expression of OPN in oxalate-treated cells. Overall, our findings suggest that vitamin E and l-arginine are candidates for further study as preventive agents for kidney stone formation. However, it would be interesting to compare these results to co-treatment of cells with oxalate and antioxidants, these studies are underway in our laboratory. Additional in vivo studies are required to confirm these in vitro results.
